# Genome-wide identification of nitrate transporter genes from *Spirodela polyrhiza* and characterization of SpNRT1.1 function in plant development

**DOI:** 10.3389/fpls.2022.945470

**Published:** 2022-08-18

**Authors:** Mengli Lv, Tiantian Dong, Jin Wang, Kaijing Zuo

**Affiliations:** ^1^Single Cell Research Center, School of Agriculture and Biology, Shanghai Jiao Tong University, Shanghai, China; ^2^Biotechnology Research Institute, Chinese Academy of Agricultural Sciences, Beijing, China

**Keywords:** nitrate transporter genes, root development, ammonium, flowering time, biomass production

## Abstract

Nitrate transporter (*NRT*) genes that participate in nitrate transport and distribution are indispensable for plant growth, development, and stress tolerance. *Spirodela polyrhiza* has the smallest genome among monocotyledon plants, and it has strong nitrate absorbance and phytoremediation abilities. However, the evolutionary history, expression patterns, and functions of the *NRT* gene family in *S. polyrhiza* are not well understood. Here, we identified 29 NRT members in the *S. polyrhiza* genome. Gene structure and phylogeny analyses showed that *S. polyrhiza* nitrate transporter (SpNRTs) genes were divided into eight clades without gene expansion compared with that in *Arabidopsis*. Transcriptomic analysis showed that *SpNRT* genes have spatiotemporal expression patterns and respond to abiotic stress. Functional analysis revealed that in *S. polyrhiza, SpNRT1.1* expression was strongly induced by treatment with nitrate and ammonium. Overexpression of *SpNRT1.1* significantly repressed primary root length, and the number and total length of lateral roots. This was more pronounced in high ammonium concentration medium. Overexpressed *SpNRT1.1* in *Arabidopsis* significantly improved biomass and delayed flowering time, indicating that the nitrate transport ability of SpNRT1.1 differs from AtNRT1.1. In conclusion, our results provide valuable information about the evolution of the NRT family in higher plants and the function of SpNRT1.1.

## Introduction

Nitrate, the most common form of nitrogen, is a limiting macronutrient for plant growth and development. Plants absorb nitrate mainly through their roots *via* nitrate transporter (NRT) proteins. Based on their nitrate affinity, NRT proteins in *Arabidopsis* can be clustered into three subfamilies. Most of the NRT1 family proteins are low-affinity nitrate transporters. AtNRT1.1 (CHL1) is the first nitrate transporter protein identified in *Arabidopsis* with high or low nitrate affinity levels switched by T101 phosphorylation states (Tsay et al., 1993; Liu et al., 1999; Liu and Tsay, 2003). NRT2 proteins are high-affinity nitrate transporters and NRT3 (also known as NAR2) proteins interact with NRT2 subfamily members to regulate nitrate uptake activity (Quesada et al., 1994; Orsel et al., 2006; Yan et al., 2011).

Plant NRT proteins are involved in numerous physiological processes and developmental stages, including tissue development (Li et al., 2017; Wang et al., 2018a), hormone (abscisic acid-ABA, gibberellins, or auxin) transport (Krouk et al., 2010; Kanno et al., 2012; Boursiac et al., 2013; Tal et al., 2016), and stress tolerance through nitrate absorbance, assimilation, and signaling pathways (Guo et al., 2003; Gojon and Gaymard, 2010; Li et al., 2010). AtNRT1.1 acts as a transceptor of nitrate, sensing environmental nitrate fluctuations and altering its nitrate transport activity (Krouk et al., 2006; Remans et al., 2006; Walch-Liu and Forde, 2008; Ho et al., 2009; Wang et al., 2009). Additionally, AtNRT1.1 is involved in nitrate-induced depolarization of guard cells; *chl1* mutant showed reduced nitrate accumulation in guard cells and reduced stomatal opening that improved drought stress tolerance in *chl1* mutant (Guo et al., 2003). AtNRT1.1 also displays auxin transport activity and mediates nitrate-modulated root development (Krouk et al., 2010; Bouguyon et al., 2015). NRT1.1 expression in *Arabidopsis* represses lateral root growth under low nitrate concentration, which is the sole nitrogen source, by promoting auxin transport out of roots (Krouk et al., 2010; Bouguyon et al., 2015), whereas AtNRT1.1 promotes lateral root formation under low nitrate condition and in the presence of ammonium (Guo et al., 2001). AtNRT1.2 has been characterized as a low-affinity nitrate transporter and an ABA transporter in *Arabidopsis* (Kanno et al., 2012; Boursiac et al., 2013). The stability and activities of NRT1.2 are regulated by the stress response, which could influence germination and vegetative growth in *Arabidopsis* (Li et al., 2020). In addition, NRTs display differences in spatiotemporal expression and nitrate transport routes in plants. NRT1.11, NRT1.12, and NRT1.7 function in the xylem-to-phloem transfer route for redistributing nitrate into development tissue (Fan et al., 2009; Hsu and Tsay, 2013); NPF2.3 is involved in nitrate translocation to shoots for acclimation to salt stress (Taochy et al., 2015). NRT1.6 expresses in the vascular tissues of the silique and funiculus for delivering nitrate into developing embryos (Almagro et al., 2008). These findings indicate that NRTs in different plant species have evolved more structural variation and functional diversity for acclimation to changing environments and stresses than expected.

*Spirodela polyrhiza* (duckweed), which belongs to the family Lemnaceae, is a fast-growing aquatic plant with a strong nutrient absorption capacity. Under optimal growth conditions, the biomass of duckweeds can double in 30 h (Wang et al., 2014). In addition, duckweed can tolerate high concentrations of ammonia and adapt to environments with a wide pH range. This makes them suitable for being widely used in phytoremediation. Genome sequence analysis showed that the size of the *S. polyrhiza* genome is 158 Mb with 19,623 protein-coding genes. *Spirodela polyrhiza* has the smallest genome among monocotyledons, being 50% smaller than that of rice (Wang et al., 2014). It has been reported that *S. polyrhiza* has a higher number of genes related to the nitrogen assimilation pathway and stronger nitrate assimilation ability than rice or *Arabidopsis* (Wang et al., 2014). To elucidate nitrate transport in *S. polyrhiza*, we analyzed *NRT* gene families in *S. polyrhiza* and characterized the spatiotemporal expression of *SpNRT*s in different tissues and under different stress conditions. We also confirmed the functions of *SpNRT1.1* and analyzed its potential to improve plant nitrogen utilization.

## Materials and methods

### Plant materials and culture conditions

Arabidopsis ecotype Columbia (Col-0) and CHL1 mutant (*chl1-5*) seeds were obtained from the Nottingham Arabidopsis Stock Centre (NASC). All seeds were sterilized with 75% ethanol for 1 min and 3% sodium hypochlorite for 10 min and plated on 1/2 Murashige and Skoog (MS) medium for germination. To analyze the effects of nitrogen concentration changes on seedling growth, MS-modified basal medium without N (Phytotechnology Laboratories)^1^ was used or supplemented with different concentrations of KNO_3_ (final concentration: 1 mm, 3 mm, or 10 mm) as the only N source. The medium contained 1.5% sucrose and 0.6% Phytoblend agar. After stratification at 4°C for 2 days, the seedlings were grown at 22°C under long-day conditions (16 h light/8 h dark) and a light intensity of 120 μmol·m^−2^ s^−1^. Root length and physiological performance of seedlings were analyzed after 8 days of growth.

*Spirodela polyrhiza* plants were kindly provided by Dr. Wang’s laboratory (Wang et al., 2014). The aseptic seedlings were transferred to liquid MS medium without nitrogen and cultivated for 3 days. The plants were then transferred to liquid medium with different concentrations of nitrogen (final concentration: 0.5 mm, 1 mm, and 5 mm KNO_3_; 0.5 mm, 1 mm, and 5 mm NH_4_Cl). The seedlings were grown under the same conditions as those of the *Arabidopsis* seedlings at 22°C.

### *SpNRT1.1* gene cloning and phylogenetic analysis

To clone the *SpNRT1.1* gene from *S. polyrhiza*, the amino acid sequences of OsNRT1.1 and AtNRT1.1 were used in the basic local alignment search tool BLASTX to search for homologous genes in the *S. polyrhiza* genome. The best-matched gene *Spipo1G0085300* (Phytozome), probably encoding SpNRT1.1 protein in *S. polyrhiza* was selected as the candidate reference sequence. To clone the full-length *SpNRT1.1* gene, the thermal asymmetric interlaced-polymerase chain reaction (Tail-PCR) method was performed using primers AD1-3 and R1-R3 (Supplementary Table 1; Liu et al., 1995; Liu and Chen, 2007). Phylogenetic analysis was performed to analyze the evolutionary relationship based on NRT1 proteins from the following nonvascular plants: *Selaginella moellendorffii*, *Physcomitrella patens*; Gymnospermae plants: *Picea abies*; and Angiospermae plants: *Vitis vinifera*, *Populus trichocarpa*, *Brachypodium distachyon*, *Oryza sativa*, *Arabidopsis thaliana*, and *S. polyrhiza*, using the maximum likelihood method by Mega X (Stecher et al., 2020).

### Subcellular localization analysis of SpNRT1.1 protein

*SpNRT1.1* CDS sequence was amplified and recombined into *pEarlyGate 101* plasmid to generate the expression cassette *CaMV35S:: SpNRT1.1-eYFP::NOS*. The *SpNRT1.1* expressing plasmid and *pEarlyGate104* plasmid was transformed into the *Agrobacterium* strain GV3101. After PCR and DNA sequencing, positive clones were selected and cultured overnight. When the cultured *Agrobacterium* reached OD_600_ = 0.6–1.0, the bacterium was centrifuged at 4000 rpm for 10 min at room temperature. The collected pellets were dissolved in a transforming solution. One-month-old tobacco leaves were infiltrated with transformation solution according to a previously reported method (Grefen et al., 2010). The infiltrated tobacco plants were kept under dark conditions for 24 h, and then transferred to normal growth conditions. The fluorescent signals were imaged by Lecia TCS SPS-II 2–3 days after infiltration.

### Generating transgenic *SpNRT1.1 Arabidopsis* plants

The open reading frame of *SPNRT1.1* was inserted into the *pHB* binary vector to generate the expression cassette *CaMV35S::SPNRT1.1-YFP::NOS*. The plasmid harboring *SPNRT1.1* gene expression cassette was then transformed into *Agrobacterium GV3101* to generate transgenic *Arabidopsis* plants. The 3–4-week-old Col-0 or *chl1-5* plants were used for flower dipping (Clough and Bent, 1998; Zhang et al., 2006). The positive plants were confirmed by hygromycin screening and PCR. Transgenic plants were self-crossed to generate T3 homozygous lines.

### NaClO_3_ uptake analysis

The seeds of *Arabidopsis* ecotype Col-0, *NRT1.1* mutant *chl1-5*, and transgenic plants (T3 homozygous lines) were sterilized with 70% ethanol for 1 min and 3% sodium hypochlorite for 10 min, and sown on 1/2 MS-modified basal medium with 1 mm NH_4_NO_3_. The seeds were stratified at 4°C for 3 days under dark conditions and then grown at 22°C under long-day conditions. 4 days later, *Arabidopsis* seedlings were transferred to 1/2 MS medium containing 2 mm NaClO_3_. The chlorophyll content and fresh weight of *Arabidopsis* seedlings were recorded after 4 days of treatment, and 6 to 10 plants were mixed as one sample. To determine the primary root length and lateral root length of *Arabidopsis* seedlings, they were photographed using a Canon camera and the measurements were calculated using ImageJ software.

### RT-qPCR analysis of gene expression

Total RNA was extracted using a Total Plant RNAprep Plant Kit (TIANGEN, China). cDNA was synthesized using the HiFiScript gDNA Removal RT MasterMix (CWBIO, China). Quantitative RT-PCR was performed according to the manual from the MagicSYBR Mixture Kit (CWBIO, China) using a LightCycler 96 System (Roche, Germany). Gene expression was calculated using the ΔΔCT method, and *SpACTIN6* (*Spipo12G0023800*) and *AtACTIN2* (Wang et al., 2018b) were used as internal controls. RT-qPCR primers used are listed in Supplementary Table 1.

### Data analysis

Student’s *t*-test were used to assess the statistical significance between the two groups. All analyses were repeated at least three times. Prism 8 software was used for the statistical analysis and to generate the diagrams.

## Results

### Genome-wide identification of *NRT* gene families in the *Spirodela polyrhiza* genome

**Figure 1:**
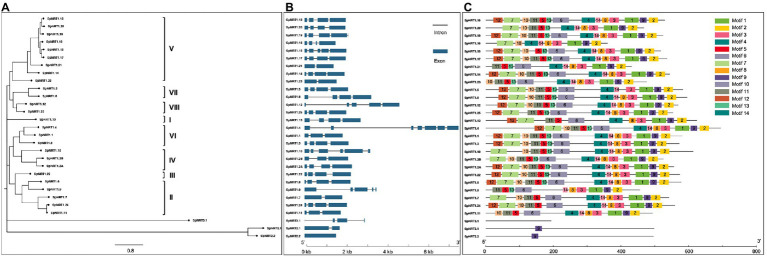
*SpNRTs* gene structure analysis and the conserved motifs in the SpNRT protein families. **(A)** Neighbor-joining method was used to construct a phylogenetic tree of SpNRT proteins by MEGA X software. All the parameters adopted the reported default values (Stecher et al., 2020). **(B)** Gene structure analysis of *SpNRT*s. The sequences of SpNRTs and gene structure information were obtained from the Ensemble plants database, and the GSDS program was used to display Gene structure (http://gsds.cbi.pku.edu.cn; Hu et al., 2015). **(C)** The predicted conserved motif of SpNRT proteins by the MEME program http://meme-suite.org/tools/meme (Bailey and Elkan, 1994; Bailey et al., 2015). TBtools was used to display the motif location (Chen et al., 2020).

To identify *NRT* gene families in *S. polyrhiza*, we used 12 functionally identified NRT1, 7 NRT2, and 2 NRT3 proteins from *Arabidopsis* and 14 NRT1, 4 NRT2, and 2 NRT3 proteins from *Oryza sativa*. BLASTP and hidden Markov models (HMM) search methods were used to search *NRT* homologs in the *S. polyhiza* genome (GeneBank Accession No.: SRX5321175). We identified 26 *NRT1* genes, 2 *NRT2* genes, and 1 *NRT3* gene from the *S. polyhiza* genome (Supplementary Table 2). Based on sequence coordinates and similarities with their homologs in *Arabidopsis*, we named all 29 *SpNRT* genes from *SpNRT1.1* to *SpNRT3.1* (Table 1). Ten *SpNRT* genes were distributed in pseudomolecules 4. Pseudomolecules 5, 8, and 22 contained two genes. Other pseudomolecules had only one gene. Two *SpNRT* genes were not assigned to any known pseudomolecules; therefore, they were labeled pseudomolecules 0. Exon/intron structure analysis showed greater structural divergence among *SpNRT* genes (Figure 1B). Most *SpNRT*s had four exons, whereas four *SpNRT* genes had two exons, three *SpNRT* genes had three exons, and three *SpNRT* genes had five exons. The difference between *SpNRT* genes in the exon/intron structure indicated that functional diversity probably existed in different NRT families.

**Table 1:** Basic information of *SpNRT* genes.

**Gene name**	**Gene ID**	**Theoretical isoeletrical point (pI)**	**MW** **(kDa)**	**Protein length**	**ORF length**	**Location coordinates**	**Intros**	**Exons**	**No. of transmembrane domain**
SpNRT1.1	Spipo1G0085300	9.07	64.90	594	1782	Pesudo1: 5346030 ~ 5,347,873	2	3	11
SpNRT1.2A	Spipo14G0009000	9.16	61.61	569	1707	Pesudo14: 527312 ~ 529,608	3	4	12
SpNRT1.2B	Spipo21G0020300	9.54	58.14	537	1,611	Pesudo21: 1257680 ~ 1,259,788	2	3	11
SpNRT1.3	Spipo5G0029200	8.46	64.22	585	1755	Pesudo5: 2200927 ~ 2,203,057	3	4	10
SpNRT1.4	Spipo8G0029500	9.00	77.65	710	2,130	Pesudo8: 2876617 ~ 2,884,084	6	7	13
SpNRT1.5	Spipo12G0038100	6.15	66.17	596	1,788	Pesudo12: 2861479 ~ 2,863,583	3	4	10
SpNRT1.6	Spipo2G0059000	9.21	63.74	591	1,773	Pesudo2: 4660756 ~ 4,662,973	3	4	10
SpNRT1.7	Spipo29G0014800	8.67	59.13	554	1,662	Pesudo29: 946495 ~ 948,323	1	2	11
SpNRT1.8	Spipo5G0063000	7.88	66.70	609	1827	Pesudo5: 5553121 ~ 5,556,355	3	4	11
SpNRT1.9	Spipo3G0094500	9.30	50.75	466	1,398	Pesudo3: 7404260 ~ 7,407,742	4	5	7
SpNRT1.10	Spipo0G0088000	9.11	69.01	627	1,881	Pesudo0: 6547962 ~ 6,551,144	5	6	10
SpNRT1.11	Spipo0G0188200	9.59	53.83	504	1,512	Pesudo0: 11521006 ~ 11,522,750	2	3	10
SpNRT1.12	Spipo4G0003200	5.70	64.03	582	1,746	Pesudo4: 222375 ~ 226,970	4	5	10
SpNRT1.13	Spipo4G0063900	8.71	70.30	638	1,914	Pesudo4: 5538377 ~ 5,541,091	3	4	12
SpNRT1.14	Spipo4G0103800	8.04	60.79	556	1,668	Pesudo4: 7798272 ~ 7,800,211	3	4	12
SpNRT1.15	Spipo4G0104400	8.03	57.36	529	1,587	Pesudo4: 7826302 ~ 7,828,322	4	5	11
SpNRT1.16	Spipo4G0104600	6.91	40.11	368	1,104	Pesudo4: 7836888 ~ 7,838,383	3	4	9
SpNRT1.17	Spipo4G0104900	7.96	59.45	548	1,644	Pesudo4: 7840223 ~ 7,842,218	3	4	9
SpNRT1.18	Spipo4G0105600	8.09	58.79	541	1,623	Pesudo4: 7861420 ~ 7,863,417	3	4	12
SpNRT1.19	Spipo4G0105800	7.08	58.34	536	1,608	Pesudo4: 7869588 ~ 7,871,714	3	4	11
SpNRT1.20	Spipo4G0106200	6.00	51.85	478	1,434	Pesudo4: 7881390 ~ 7,883,362	3	4	10
SpNRT1.21	Spipo4G0106600	7.59	48.56	441	1,323	Pesudo4: 7886885 ~ 7,888,290	1	2	7
SpNRT1.22	Spipo15G0031400	8.92	63.99	587	1761	Pesudo15: 3080060 ~ 3,082,306	3	4	11
SpNRT1.23	Spipo17G0047200	9.67	53.44	486	1,458	Pesudo17: 3358024 ~ 3,359,573	1	2	9
SpNRT1.24	Spipo22G0027500	8.95	61.22	572	1716	Pesudo22: 2022278 ~ 2,024,329	3	4	11
SpNRT1.25	Spipo22G0043000	8.47	62.85	566	1,698	Pesudo22: 2855519 ~ 2,857,536	3	4	10
SpNRT2.1	Spipo8G0057400	8.65	55.25	509	1,527	Pesudo8: 4572055 ~ 4,573,747	1	2	11
SpNRT2.2	Spipo19G0017400	6.90	53.66	507	1,521	Pesudo19: 1264026 ~ 1,265,549	0	1	10
SpNRT3.1	Spipo13G0039300	9.65	21.14	197	591	Pesudo13: 2637713 ~ 2,640,626	3	4	1

The pI and Mw were calculated using ExPASY (http://web.expasy.org/compute_pi/). The transmembrane domains of the NRT proteins were predicted by accessing TMHMM 2.0 online (http://www.cbs.dtu.dk/services/TMHMM/).

To identify the evolutionary relationship between SpNRTs and other homologous proteins, we constructed a multi-species phylogenetic tree using the maximum likelihood method based on 582 NRT proteins from *Volvox carteri*, *Selaginella moellendorffii*, *Physcomitrella patens*, *Picea abies*, *Vitis vinifera*, *Populus trichocarpa*, *Brachypodium distachyon*, *Oryza sativa, Arabidopsis thaliana*, and *S. polyhiza*. All 582 proteins were divided into three subfamilies, of which 516 proteins belonged to the NRT1 subfamily, 48 proteins belonged to the NRT2 subfamily, and 18 proteins belonged to the NRT3 subfamily (Supplementary Figure 1; Supplementary Table 3). SpNRT1 subfamilies were further divided into eight clades: Clade I (one protein), Clade II (five proteins), Clade III (one protein), Clade IV (three proteins), Clade V (nine proteins), Clade VI (three proteins), Clade VII (two proteins), and Clade VIII (two proteins; Figure 1A). Protein structure analysis indicated that each NRT subfamily had similar conserved motifs and transmembrane domains (TM), similar to homologous proteins from *Arabidopsis*. The number of TM in SpNRT1 proteins ranged from 7 to 13; SpNRT2 proteins had 10 or 11 TMs, whereas SpNRT3.1 had only 1 TM. The conserved TM domain structure in SpNRTs indicated that they might have nitrate transport ability similar to that of the orthologs in *Arabidopsis*.

Amino acid alignment of SpNRTs identified 14 motifs among SpNRT proteins, 13 of which were located in the TM areas of SpNRT1 proteins (Figure 1C; Supplementary Figure 2). Motif 1 is located in the TM10 and TM 10-TM 11 loops, in which the 34th amino acid is usually P, and its mutation can result in the loss of nitrate transporter function of AtNRT1.1 (Supplementary Figures 3, 4). Motif 4 was located in TM 7, in which the 24th amino acid is the nitrate-binding site affecting the nitrate uptake efficiency of NRT1.1 in maize (Sun et al., 2014; Wen et al., 2017). Motif 6 with interface residues was located in TM 5, TM 5-TM 6 loop, or TM6. Motif 10 was located in the TM 3 or TM 3-TM 4 loop, where the 21st amino C forms a disulfide linkage with the 5th C in motif 11. Motif 12, with the 20th-ExxERFxYY-28th motif, is functional in proton coupling (Supplementary Figures 3, 4). SpNRT3 had no motif similar to SpNRT1s and SpNRT2s. The SpNRT2 subfamily had a motif (motif 9) similar to SpNRT1s. Motif 9 is usually located from the 150th to 170th amino acid in SpNRT2.1 or from the 139th to 159th amino acid in SpNRT2.2, in which Phe^511^ could form a nitrate-binding pocket in AtNRT1.1 (Sun et al., 2014). These changes in the motif structure indicate that SpNRTs have their own characteristics in nitrate transport.

### Expression patterns of *SpNRTs* in different tissues and under abiotic stresses

**Figure 2:**
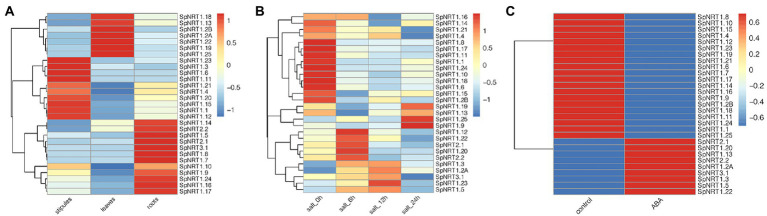
Expression patterns of *SpNRT* genes. **(A)** Relative expression level of *SpNRT* genes in various tissues determined by RNA-seq FPKM value. **(B)** Relative expression level of SpNRT genes response to salt stress determined by RNA-seq FPKM value. **(C)** Relative expression level of SpNRT gene response to ABA stress determined by RNA-seq FPKM value. Red means relative expression high and blue means lower. SpNRTs RNA-seq data downloaded from NCBI Sequence Read Archive (SRA) database were used to express profile analysis, all the data is listed in Supplementary Tables 4–7. FPKM (fragments per kilobase of transcript per million fragments mapped) value standardized by the Z-score method of every SpNRT gene was used to generate heatmaps by TopHat and Cuffilinks software.

To analyze the expression patterns of *SpNRTs* in different tissues, we used RNA-seq data from leaves, stipules, and roots of *S. polyrhiza* to compare their transcriptional levels (Supplementary Tables 4, 5). The expression patterns of the 29 *SpNRT* genes were classified into three groups (Figure 2A). Group I was highly expressed in the leaves and contained seven genes, including *SpNRT1.18*, *SpNRT1.13*, *SpNRT1.2A*, *SpNRT1.2B*, *SpNRT1.22*, *SpNRT1.19*, and *SpNRT1.25*. Group II genes with higher expression in stipules included the following ten *NRT* genes: *SpNRT1.23*, *SpNRT1.3*, *SpNRT1.6*, *SpNRT1.11*, *SpNRT1.21*, *SpNRT1.4*, *SpNRT1.20*, *SpNRT1.15*, *SpNRT1.1*, and *SpNRT1.12*. Group III genes were highly expressed in roots, these were *SpNRT1.14*, *SpNRT1.5*, *SpNRT1.7*, *SpNRT1.8*, *SpNRT1.9*, *SpNRT1.16*, *SpNRT1.17*, *SpNRT1.24*, *SpNRT2.1*, *SpNRT2.2*, *SpNRT3.1*, *SpNRT2.1*, *SpNRT2.2*, and *SpNRT3.1*. This suggests that *SpNRT3.1* may have the same function as *AtNRT3.1* in *Arabidopsis*, in which SpNRT3.1 cooperates with SpNRT2s in nitrate transport. The difference in expression in different tissues indicated that SpNRTs function in different plant growth processes across tissues.

Duckweeds are widely recognized in phytoremediation with a high tolerance to abiotic stresses. We further analyzed the expression patterns of *SpNRTs* in response to salt stress (Figure 2B; Supplementary Tables 4, 6). The expression of *SpNRTs* in Group I was strongly inhibited by salt stress treatment, which included 14 (48.3%) NRT genes and 6 from the NPF5 group (9 *NPF5* genes in *S. polyrhiza,* Figure 1A). The expression of *SpNRT* genes in Group II was initially inhibited by salt stress after 6 h or after 12 h; thereafter, the expression increased significantly. Group II contained four *SpNRTs* from four different groups. In contrast to Group I and II, the expression of *SpNRTs* in Group III was clearly induced by salt stress at 6 h but significantly decreased after 24 h of treatment. Group III contained 10 genes, including all genes from the *NRT2* and *NRT3* subfamilies. Interestingly, the expression of *SpNRTs* showed two completely different responses to ABA treatment, either strongly increasing or decreasing after ABA treatment (Figure 2C; Supplementary Tables 4, 7), indicating that *SpNRTs* likely transport or redistribute nitrate under different abiotic stress conditions.

### *SpNRT1.1* gene cloning and phylogenetic analysis

NRT1.1 is an important nitrate sensor and nitrate transporting protein in *Arabidopsis*; therefore, we cloned the full-length cDNA of *SpNRT1.1* gene by Tail-PCR and analyzed its function. *SpNRT1.1* gene encodes a protein with 594 amino acids and a theoretical isoelectric point of 9.07. SpNPF6 subfamily proteins aligned with NPF6 proteins from gymnosperms and angiosperms, indicating that SpNRT1.1 was close to OsNRT1.1A with 70% similarity. The phylogenetic tree showed that the NPF6s from nonvascular plants, including *Selaginella tamariscina* and *Physcomitrella patens*, were clustered into one independent clade with the greatest genetic distance from other NPF6s (Supplementary Figure 5). The subcategory number of NPF6 proteins tended to show increased levels in angiosperms relative to gymnosperms. No NRT1.1 homolog was identified in gymnosperms, but NRT1.1, NRT1.3, and NRT1.4 subcategories were found in angiosperms. The conserved domain showed that SpNRT1.1 has 12 TMs, the conserved phosphorylation site is T102 on TM3, and the T101 site corresponds to a functional switch from high-affinity to low-affinity nitrate transport in *Arabidopsis* (Supplementary Figure 3). The NO_3_^−^ binding site on TM7 (Y353) in SpNRT1.1 was similar to that in OsNRT1.1A and OsNRT1.1C but different from those of AtNRT1.1 and OsNRT1.1B (Supplementary Figure 4). The structure of SpNRT1.1 indicated that the nitrate transporting activity of SpNRT1.1 may be different from that of AtNRT1.1.

### SpNRT1.1 protein is localized on the plasma membrane

**Figure 3:**
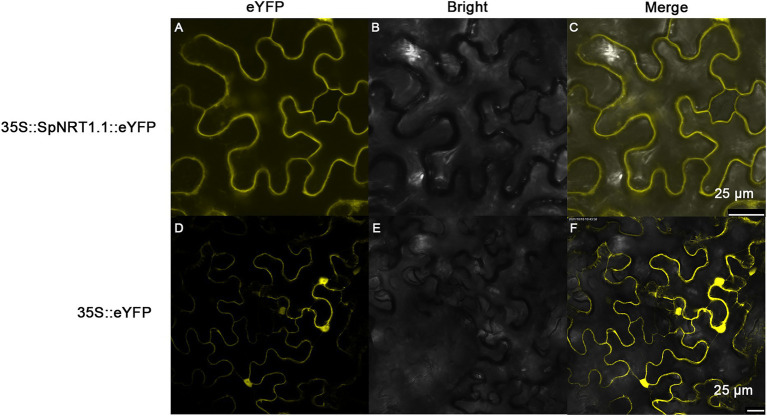
Localization of SpNRT1.1 protein in the plasma membrane of tobacco epidermal leaves. The CDS sequence of *SpNRT1.1* gene fused with the eYFP sequence under the control 35S promoter was transiently expressed in tobacco leaves **(A–C)**, using the eYFP protein as the control **(D–F)**. **(A,D)** eYFP fluorescence; **(B,E)** bright field images; **(C,F)** merged eYFP fluorescence with bright field. Scale bar = 25 μm.

To analyze the subcellular localization of the SpNRT1.1 protein, SpNRT1.1-eYFP fusion protein was transiently expressed in the epidermal cells of tobacco leaves. The fluorescence signals of SpNRT1.1-eYFP protein were found on the plasma membrane, compared with eYFP signals appearing in the nucleus, cytoplasm, and cell membrane (Figure 3), indicating that SpNRT1.1 is a transmembrane protein.

### *SpNRT1.1* gene expression induced by nitrate and ammonium in *Spirodela polyrhiza*

To analyze the expression patterns of *SpNRT1.1* gene in *S. polyrhiza*, we transferred cultured duckweed plants grown in a nitrogen-free medium into the liquid 1/2 MS medium with different concentrations of nitrogen (0.5 mm, 1 mm, and 5 mm NO_3_^−^; or 0.5 mm, 1 mm, and 5 mm NH_4_^+^). RT-qPCR analysis showed that *SpNRT1.1* expression was induced by both nitrate and ammonium, with transcription levels reaching the peak 30 min after treatment under both low and high concentrations (Supplementary Figure 6). Compared with the *AtNRT1.1* expression in Col-0 plants, the *SpNRT1.1* transcripts were 100 times higher than that of *AtNRT1.1* in Arabidopsis (Supplementary Figure 6C), indicating that Sp*NRT1.1* is functional in both ammonium and nitrate in *S. polyrhiza*, which is completely different from *AtNRT1.1* in Arabidopsis.

### *SpNRT1.1* complements the phenotype of *chl1-5* mutant in arabidopsis under chlorate-supplied conditions

**Figure 4:**
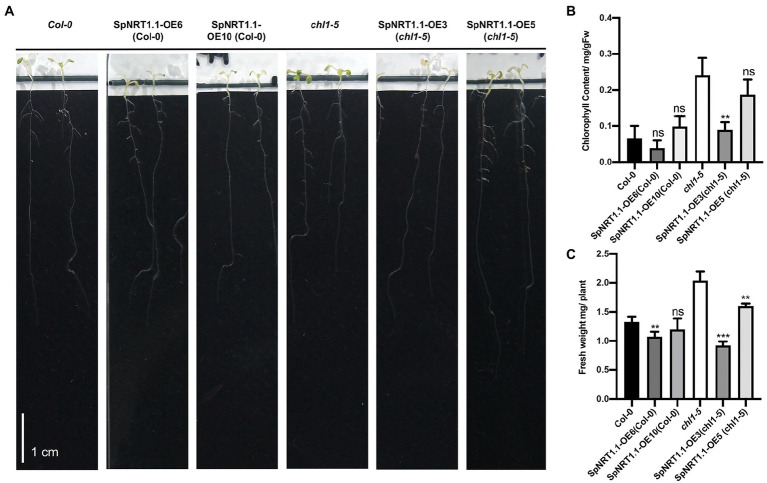
Overexpression of *SpNRT1.1* in Col-0 and *chl1-5* in Arabidopsis leads to etiolated phenotype under chlorate-supplied conditions. **(A)** Phenotype of *SpNRT1.1* overexpressed lines and complemented lines under chlorate-supplied conditions. **(B)** Chlorophyll content of Col-0 and transgenic lines compared with *chl1-5* plant. **(C)** Shoot fresh weight of Col-0 and transgenic lines compared with *chl1-5* plant. Student’s *t*-test was used for significant difference analysis. Asterisks indicate significance levels compared with *chl1-5* mutant plants, *n* ≥ 3, ^**^p < 0.01, ^***^p < 0.001. Scale bar = 1 cm.

ClO_3_^−^ can be reduced by nitrate reductase to chlorite, which is toxic to plants, resulting in etiolated leaves of *chl1-5* mutant plants. Therefore, it can be used for nitrate uptake analysis (Tsay et al., 1993; Huang et al., 1999). To check whether SpNRT1.1 functions in the uptake of ClO_3_^−^, 2 mm ClO_3_^−^ was added to 1/2 MS medium to treat *chl1-5* mutant plants which had been complemented by *SpNRT1.1*. Chlorophyll content and fresh weight of shoots were significantly decreased in both Col-0 and *SpNRT1.1* complemented plants and *SpNRT1.1* overexpressed plants compared with *chl1-5* mutant plants (Figure 4). These results suggest that SpNRT1.1, which functions in nitrate absorption, is sensitive to chlorate.

### Overexpressed *SpNRT1.1* gene in Arabidopsis represses root development

**Figure 5:**
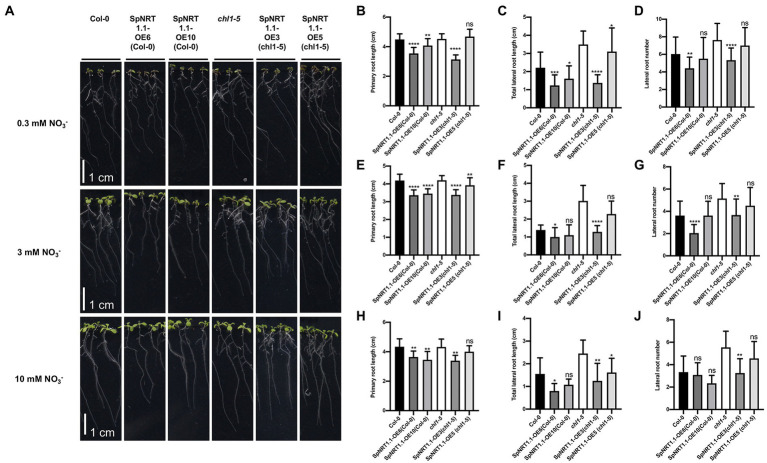
Overexpression of *SpNRT1.1* in Col-0 and *chl1-5* plants suppress primary and lateral root growth under different nitrate concentrations. **(A)** Phenotype of *SpNRT1.1* overexpression lines and complemented lines under different nitrate-supplied concentrations. **(B–J)** Primary root length, lateral root number, and total lateral root lengths of *SpNRT1.1* overexpression lines and complemented plants under 0.3 mm NO_3_^−^-supplied condition **(B–D)**, 3 mm **(E,F)**, 10 mm **(H–J)**. Student’s *t*-test was used for significant difference analysis. Asterisks indicate significance levels compared with *chl1-5* mutant plants, ^*^p < 0.05, ^**^p < 0.01, ^***^p < 0.001, ^****^p < 0.0001. *n* ≥ 10, Scale bar = 1 cm.

To analyze the function of SpNRT1.1 in Arabidopsis, we analyzed the root phenotypes of Col-0 and *chl1-5* with ectopically expressed *SpNRT1.1* in plants growing in 1/2 MS medium under different concentrations of nitrate (0.3 mm, 3 mm, and 10 mm KNO_3_; Figure 5A). Primary root length of *SpNRT1.1* overexpressing plants slightly decreased (Figures 5B,E,H); the total lateral root length (Figures 5C,F,I) and lateral root number (Figures 5D,G,J) of *SpNRT1.1* overexpressing plants were significantly repressed compared to those of Col-0 and *chl1-5* plants after 8 d of growth. These results indicate that overexpression of *SpNRT1.1* in Arabidopsis has negative effects on root growth.

**Figure 6:**
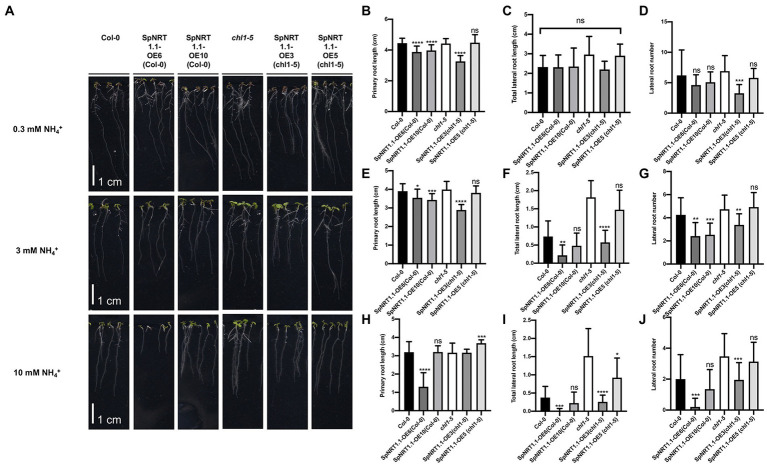
Overexpression of *SpNRT1.1* in Col-0 and *chl1-5* in *Arabidopsis* suppresses primary and lateral root growth under different ammonium concentrations. **(A)** Phenotype of *SpNRT1.1* overexpressed lines and complementary lines under different ammonium-supplied concentrations. **(B–J)** Primary root length, lateral root number, and total lateral root length of *SpNRT1.1* overexpressed and complementary plants under 0.3 mm NH_4_^+^-supplied condition **(B–D)**, 3 mm **(E,F)**, 10 mm **(H–J)**. Student’s *t*-test was used for significant difference analysis. Asterisks indicate significance levels compared with *chl1-5* mutant plants, ^*^p < 0.05, ^**^p < 0.01, ^***^p < 0.001, ^****^p < 0.0001, *n* ≥ 10, Scale bar = 1 cm.

AtNRT1.1 can regulate ammonium absorbance, leading to pH and auxin changes in roots, and *AtNRT1.1* mutant plants are tolerant to ammonium/low pH in the growing medium (Hachiya and Noguchi, 2011; Meier et al., 2020). To analyze the effects of *SpNRT1.1* on root growth, Col-0, *chl1-5*, and *SpNRT1.1* overexpressing plants were grown in 1/2 MS medium with different concentrations of ammonium. Under conditions of high concentrations of ammonium (3 mm and 10 mm ammonium), primary root length and the number of lateral roots in the *SpNRT1.1* overexpressing lines were significantly decreased (Figure 6), indicating that SpNRT1.1 also functions in ammonium absorbance.

**Figure 7:**
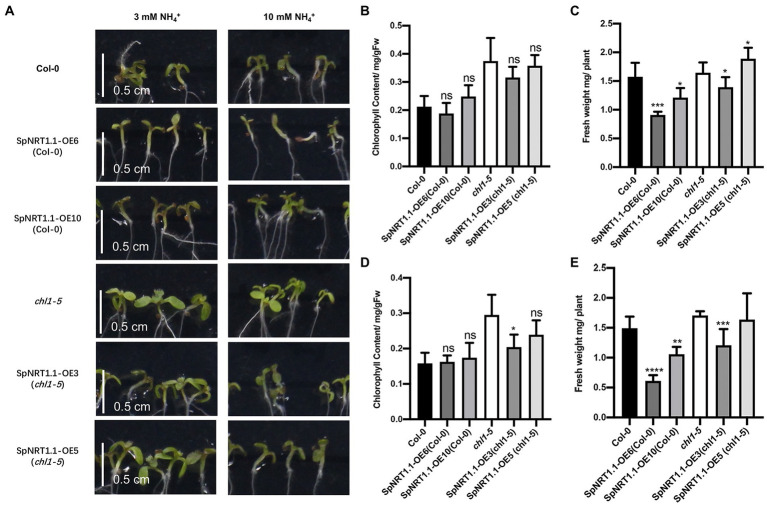
Overexpression of *SpNRT1.1* in Col-0 and *chl1-5* plants leads to sensitivity to ammonium. **(A)** Phenotype of *SpNRT1.1* overexpressed and complemented plants under different ammonium concentrations. Chlorophyll content and fresh weight under 3 mm NH_4_^+^ **(B,C)** and 10 mm NH_4_^+^ **(D,E)**. Student’s *t*-test was used for significant difference analysis. Asterisks indicate significance levels compared with *chl1-5* mutant plants, ^*^p < 0.05, ^**^p < 0.01, ^***^p < 0.001, ^****^p < 0.0001, *n* ≥ 5, Scale bar = 0.5 cm.

To further analyze whether SpNRT1.1 has a similar function in ammonium tolerance (Hachiya and Noguchi, 2011), we measured the chlorophyll content and fresh weight of *SpNRT1.1* complemented plants and Col-0 plants in the medium containing 3 and 10 mm ammonium, respectively. The chlorophyll content and fresh weight of *SpNRT1.1* complemented and Col-0 plants were decreased compared with those of *chl1-5* mutant plants (Figure 7); they were found to decrease with the increase in ammonium concentration in the medium. The chlorophyll content and fresh weight of *SpNRT1.1* complemented plants decreased slightly compared with Col-0 plants. These results suggest that SpNRT1.1 has a similar but weaker sensitivity to ammonium compared with AtNRT1.1 protein.

### Overexpression of *SpNRT1.1* in *Arabidopsis* delayed flowering and increased biomass production

**Figure 8:**
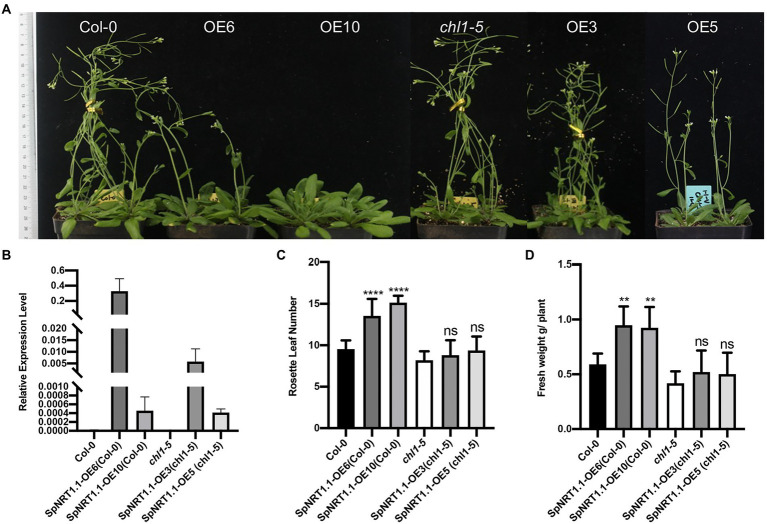
Overexpression of *SpNRT1.1* in Col-0 and *chl1-5* plants leads to late flowering time and improved biomass. Ordinary one-way ANOVA was used for statistical significance analysis, *n* ≥ 5, *p* < 0.05. **(A)** Phenotype of *SpNRT1.1* overexpressed and complemented plants. **(B)** Relative expression level of *SpNRT1.1* gene. Expression data display in Supplementary Table 8. **(C)** Rosette leaf number and **(D)** Fresh weight of *SpNRT1.1* overexpressed and complemented plants. Student’s *t*-test was used for significant difference analysis. Asterisks indicate significance levels compared with *chl1-5* mutant plants, ^**^p < 0.01, ^****^p < 0.0001, *n* ≥ 5.

To analyze the effect of *SpNRT1.1* overexpression on plant growth, Col-0 and *SpNRT1.1* overexpressing plants were grown in vermiculite soil with 10 mm nitrate. After 1 month of growth, the flowering time of SpNRT1.1 overexpressing lines was delayed by approximately 5 days, gene expression analysis that SpNRT1.1 overexpression repressed the transcription levels of *SOC1* gene, which directly activates floral identity gene LEAFY (Lee and Lee, 2010). Reproduction transition gene *FT* and *FD* gene expression were repressed to some extent by *SpNRT1.1* overexpression (Supplementary Figure 7). And their biomass increased by approximately 60% compared with Col-0 plants (Figure 8), indicating that SpNRT1.1 expression influences nutrition absorbance and maturation in *Arabidopsis*.

## Discussion

*Spirodela polyrhiza*, a duckweed, is the smallest, fast-growing, and morphologically simplest flowering plant. The rapid growth rate of *S. polyrhiza* requires sufficient nutrients, including nitrogen and phosphorus, to maintain its development and normal life cycle. Many *S. polyrhiza* protein families have been identified such as the lipoxygenase gene family (Upadhyay et al., 2020), WRKY gene family (Zhao et al., 2021), and polyamine biosynthesis pathway (Upadhyay et al., 2021). NRT proteins act as transporters that acquire nitrogen from the environment. However, there is limited knowledge regarding the evolution, protein structure, and function of NRT families in *S. polyhiza*. The *SpNRTs* gene family was identified in this study using BLASTP and HMM search methods. A total of 29 NRT genes were identified from the *S. polyrhiza* genome, and they were divided into three NRT subfamilies: 26 NPF (NRT1/PTR, NPF), 2 NRT2, and 1 NRT3. Compared with NRT proteins in the rice genome, *S. polyrhiza* has fewer NRT1 family members. The stronger nitrate absorbance ability of *S. polyrhiza* does not result from the expansion of NRT proteins during evolution compared with other monocotyledons. For example, *S. polyrhiza* has only one *NRT1.1* gene, whereas rice has four NRT1.1 members (*NRT1.1A, NRT1.1B, NRT1.1C*, and *NRT1.1D*), and maize has four NRT1.1 genes. SpNRT1.1 s possess low identity with their counterparts from Arabidopsis (61%) and rice (70%). This indicates that the SpNRT1 protein may have a different protein structure and nitrate transport ability from NRT1 in Arabidopsis or rice.

Conserved domain analysis showed that SpNRTs contain all the conserved TM domains and motifs in the NRTs of *Arabidopsis*, and motif 9 specifically exists in SpNRT1 and SpNRT2 proteins. The 10th amino acid of motif 9 is the hydrophobic residue Phe^511^, which, in association with Leu^49^, Val^53^, and Leu^78^ can form a nitrate-binding pocket similar to AtNRT1.1. This result indicated that motif 9 in SpNRT1s and SpNRT2s is probably involved in nitrate uptake activity. In addition, SpNRT1 proteins also have conserved amino acid residues responsible for nitrate transport in AtNRT1.1 (the 24th amino acid in Motif4, and the 34th amino acid in motif 1). Taken together, these results indicate that SpNRT1.1 has nitrate transport ability, and its activities are slightly different from those of AtNRT1.1.

NRT1.1 is the most important nitrate transporter and receptor. It is identified and characterized in various species. NRT1.1 members in different plant species have different nitrate absorbance and nitrate signal sensing characteristics. Rice OsNRT1.1A is a low-affinity nitrate transporter, and overexpression *OsNRT1.1A* in rice improves rice yield and significantly promotes early maturation (Wang et al., 2018b). OsNRT1.1B is a constitutive nitrate transporter, and overexpression of *OsNRT1.1B* increased nitrogen accumulation under both low and high nitrate conditions, but *OsNRT1.1A* increased nitrogen accumulation in rice only under high nitrate conditions (Fan et al., 2016). Maize has 4 *NRT1.1* genes; *ZmNRT1.1A-D*, of which ZmNRT1.1A (ZmNPF6.4) is a low-affinity nitrate transporter, and ZmNRT1.1B (ZmNPF6.6) is a pH-dependent non-biphasic high-affinity nitrate transporter (Plett et al., 2010; Wen et al., 2017; Wang et al., 2020). These diverse characteristics prompted us to investigate the functions of *SpNRT1.1*.

*SpNRT1.1* expression was strongly induced by nitrate and ammonium. This inducible expression pattern is similar to that of *OsNRT1.1A*, indicating that *SpNRT1.1* is probably involved in the rapid ammonium utilization in aquatic plants (Wang et al., 2018b). The *chl1-5* plants complemented by *SpNRT1.1* displayed decreased ammonium toxicity compared with Col-0 and SpNRT1.1 overexpression lines. We deduced that *SpNRT1.1* has a greater ammonium tolerance than *AtNRT1.1* (Hachiya and Noguchi, 2011), which benefits the growth of *S. polyrhiza* in water condition. The biomass and flowering time of *SpNRT1.1* overexpressing plants were drastically changed compared with those of Col-0. However, the phenotypes of SpNRT1.1 complemented lines were slightly changed. One probable reason for the inconsistency between the phenotype of SpNRT1.1 overexpression lines and SpNRT1.1 complemented lines was that SpNRT1.1 and AtNRT1.1 had the different sensitivity to ammonium or pH and different genetic backgrounds. Additionally, SpNRT1.1 and AtNRT1.1 proteins form heterodimers, and the function of SpNRT1.1-AtNRT1.1 heterodimers differs from that of AtNRT1.1-AtNRT1.1 homodimers. In *Arabidopsis*, nitrate treatment can change the phosphorylation profile of NRT1.1 mutant plants, including the auxin transporter PIN2, (Vega et al., 2021). Auxin accumulation depressed root growth (Li et al., 2015), and many auxin transporter induced by nitrate, such as PIN1, PIN2, PIN3, PIN4, PIN7, AUX1, LAX3 and so on (Maghiaoui et al., 2020). Auxin transporter *AUX1, LAX3* and auxin signaling F-box 3 (AFB3) gene expression was induced by SpNRT1.1 (Supplementary Figure 8), mutation of AUX1 leads to increased root length under high nitrogen condition (Li et al., 2015), LAX3 or AFB3 probably play the similar role with AUX1, although they positively regulated root growth under low nitrogen condition. CLV1 repressed lateral root emergence (Liu et al., 2020), which expression induced in our experiment. These indicted SpNRT1.1 negative regulated root growth under high nitrogen cultured condition. Local ammonium promotes lateral root emergence to develop a highly branched root system, as ammonium acidifies the root apoplast and increases auxin content in the cortex and epidermis (Meier et al., 2020). This suggests that ectopic expression *SpNRT1.1* in *Arabidopsis* improved nitrogen absorbance and prolonged vegetative growth by affecting auxin transport. How SpNRT1.1 regulates root structure and ammonium tolerance through auxin transduction needs to be determined in future studies.

To conclude, we identified all NRT proteins in *S. polyrhiza* at the genomic level and analyzed their conserved domains and motifs. Transcriptomic analysis showed that SpNRTs had spatiotemporal expression patterns and quickly responded to various abiotic stresses, indicating their complex and diverse functions in nitrate uptake and stress tolerance. Functional analysis of *SpNRT1.1* in *Arabidopsis* presented the potential use of SpNRT1.1 in increasing crop biomass and should be extensively studied in future. Future studies should determine why SpNRT1.1 had a different function in case of sensitivity towards ammonium and whether the conserved T102 is an important phosphorylation site similar to T101 in AtNRT1.1.

## Data availability statement

The datasets presented in this study can be found in online repositories. The names of the repository/repositories and accession number(s) can be found in the article/Supplementary material.

## Author contributions

KZ and ML designed and conducted the experiments. ML and TD performed the experiments. ML analyzed the data. ML, JW, and KZ wrote and revised the article. All authors contributed to the article and approved the submitted version.

## Funding

This work was supported by National Key R&D Program of China (No. 2018YFA0901000 and 2018YFA0901003). We also appreciate the support of BIO-Agri project of SJTU and the Agricultural Science and Technology Innovation Program of CAAS.

## Conflict of interest

The authors declare that the research was conducted in the absence of any commercial or financial relationships that could be construed as a potential conflict of interest.

## Publisher’s note

All claims expressed in this article are solely those of the authors and do not necessarily represent those of their affiliated organizations, or those of the publisher, the editors and the reviewers. Any product that may be evaluated in this article, or claim that may be made by its manufacturer, is not guaranteed or endorsed by the publisher.

## Footnotes

^1^ https://phytotechlab.com

## Abbreviations

ANOVA: Analysis of variance
BLAST: Basic local alignment search tool
HMM: Hidden Markov models
NRT: Nitrate transporter
MS medium: Murashige and Skoog medium
PCR: Polymerase chain reaction
SpNRTs: *S. polyrhiza* nitrate transporter
TM: Transmembrane domains
