# Supplementary material for: Genome-wide identification of nitrate transporter genes from Spirodela polyrhiza and characterization of SpNRT1.1 function in plant development
Source: Front Plant Sci. 2022 Aug 18;13:945470. doi: 10.3389/fpls.2022.945470 (PMC9436390; doi:10.3389/fpls.2022.945470)
Supplement: Supplementary file 2 [file Data_Sheet_2.docx]

Supplementary Figure


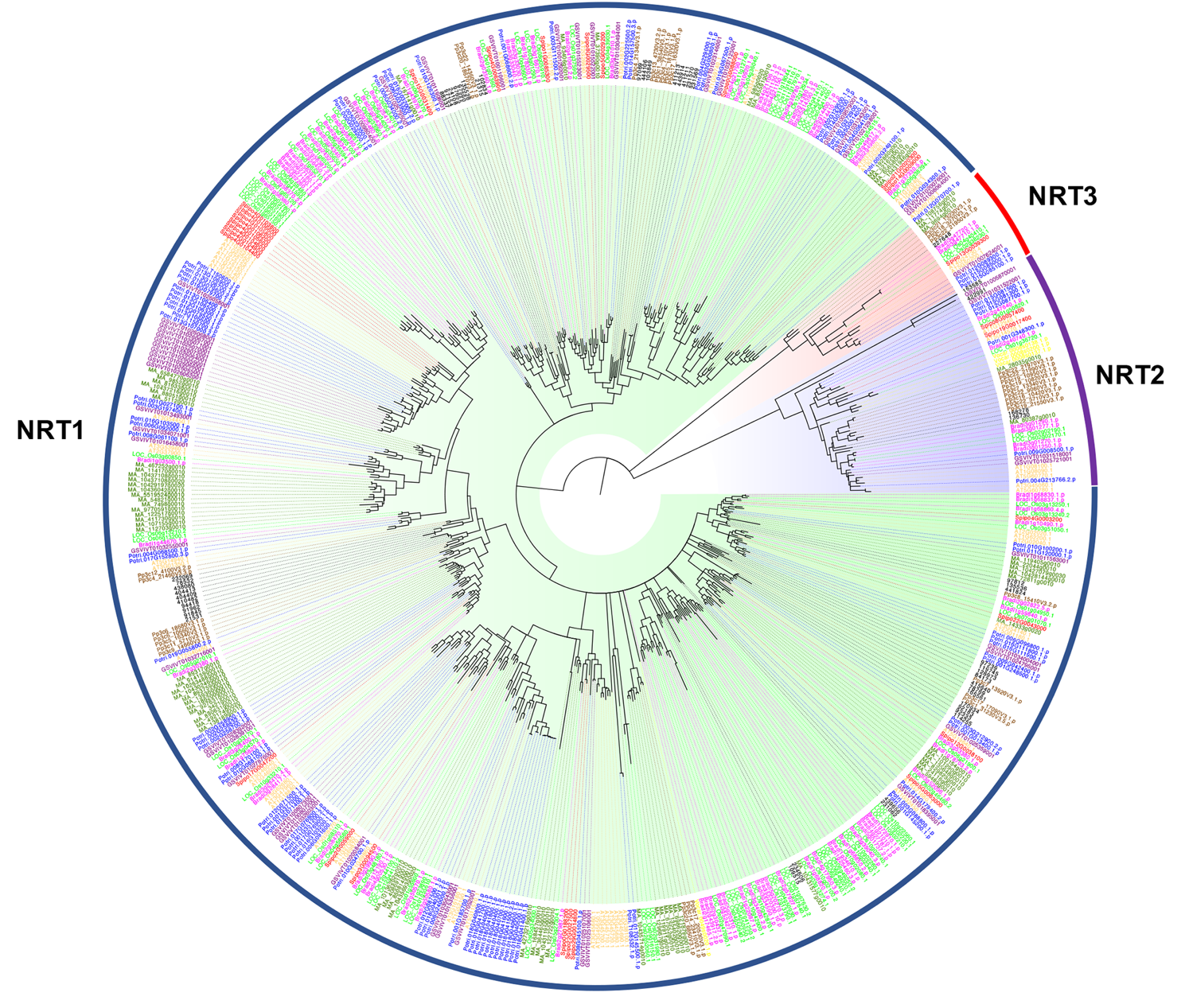


**Supplementary Figure 1. Phylogenetic tree of NRTs from *Volvox carteri*, *Selaginella moellendorffii*, *Physcomtrella patens*, *Picea abies*, *Vitis vinifera*, *Populus trichocarpa*, *Brachypodium distachyon*, *Oryza sativa,* *Arabidopsis* *thaliana* and *Spirodela polyhiza.***

Maximum Likelihood method was used to generate phylogenetic trees of NRT1, NRT2 and NRT3 protein. NRTs in phylogenetic tree from different species in different colors. *Volvox carteri* in light yellow, *Selaginella mollendorffii* in black, *Physcomtrella patens* in brown, *Picea abies* in bottle green, *Vitis vinifera* in purple, *Populus trichocarpa* in blue, *Brachypodium distachyon* in rose red, *Oryza sativa* in light green, *Arabidopsis thaliana* in orange and *Spirodela polyhiza* in red.


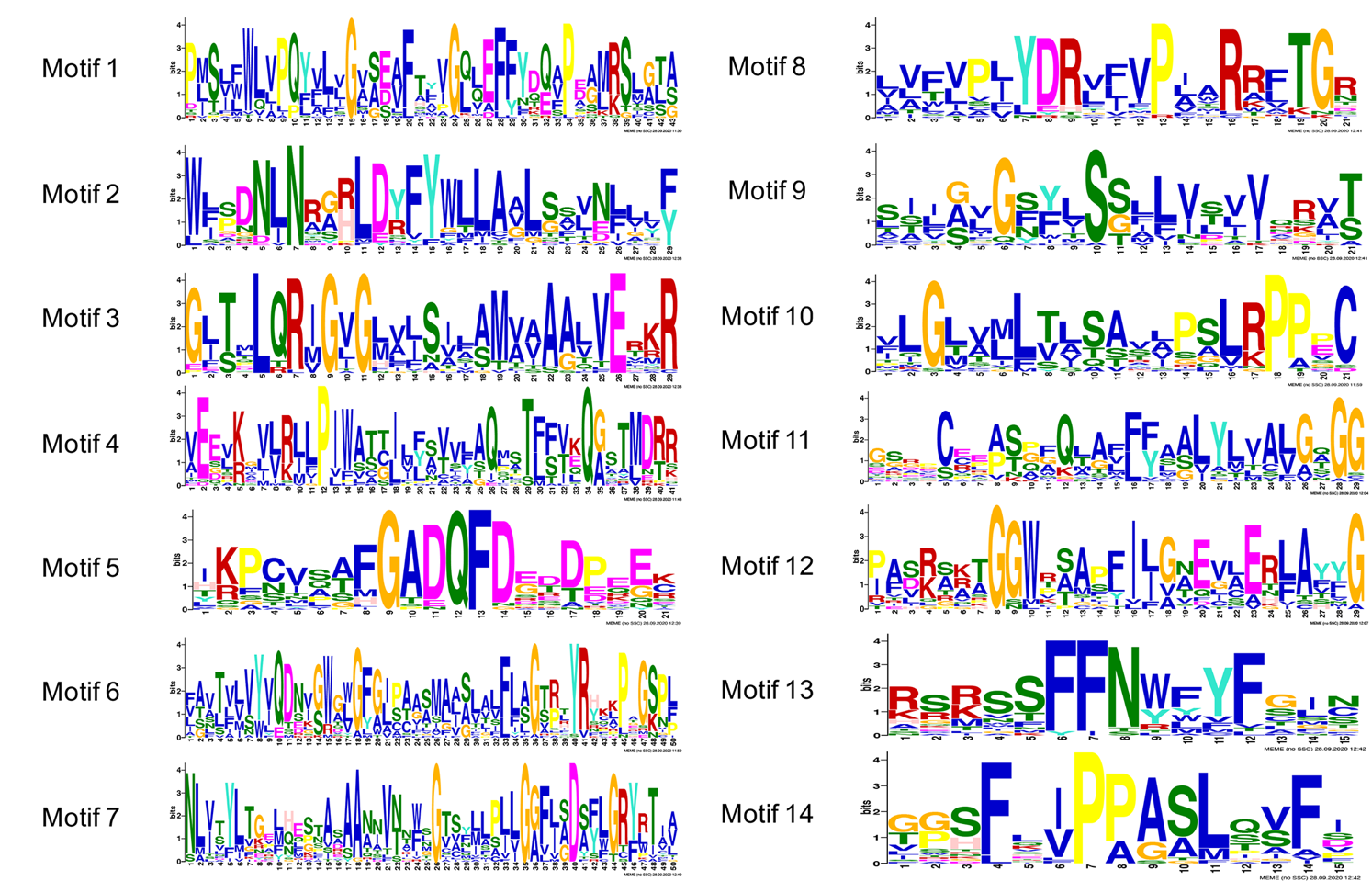


**Supplementary Figure 2. Conserved motif of SpNRTs in *Spirodela polyhiza.***

The predicted conserved motif on SpNRT proteins by MEME program <http://meme-suite.org/tools/meme> (Bailey et al., 1994, 2015).


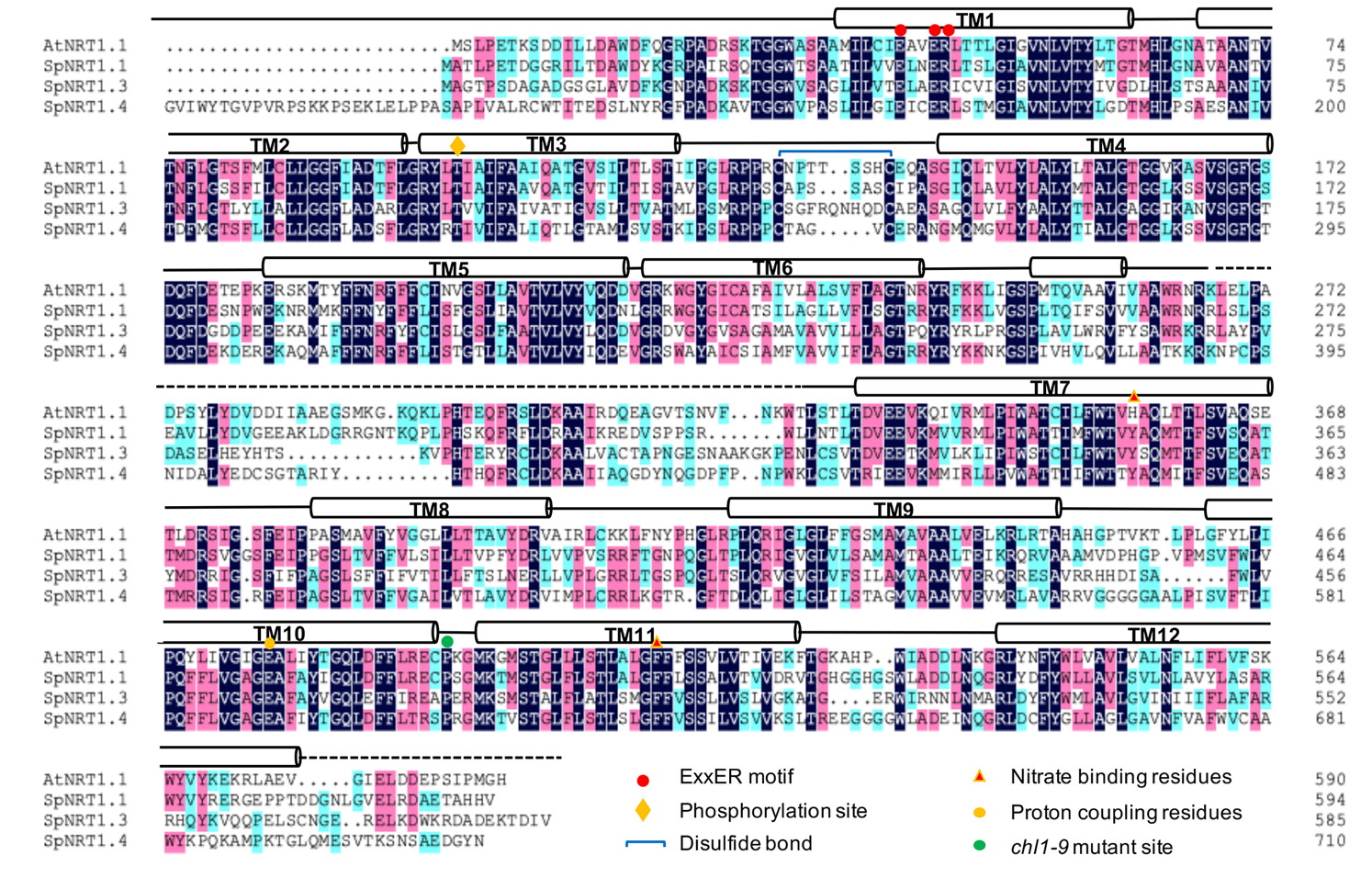


**Supplementary Figure 3. Multiple alignment of SpNPF6 in *Spirodela polyhiza.***

SpNRT1.1, SpNRT1.3, SpNRT1.4 and AtNRT1.1 protein sequence were used for sequence alignment by DNAMAN software. The identical residues were shaded in different colors according to identity score, 100% in dark blue, ≥75% in red, ≥50% in wathet blue, <33% in white. The key amino site for NRT1.1 function were marked out according to the research of Sun et al (2014). The putative transmembrane domains (TM) are marked with black column and are numbered. The putative ExxER motif marked with red dot. The phosphorylation site corresponding to T101 on AtNRT1.1 marked with orange diamond. The putative disulfide bond marked with blue square brackets. The putative proton coupling residue marked with orange dot. The putative nitrate binding residues marked with red triangle.


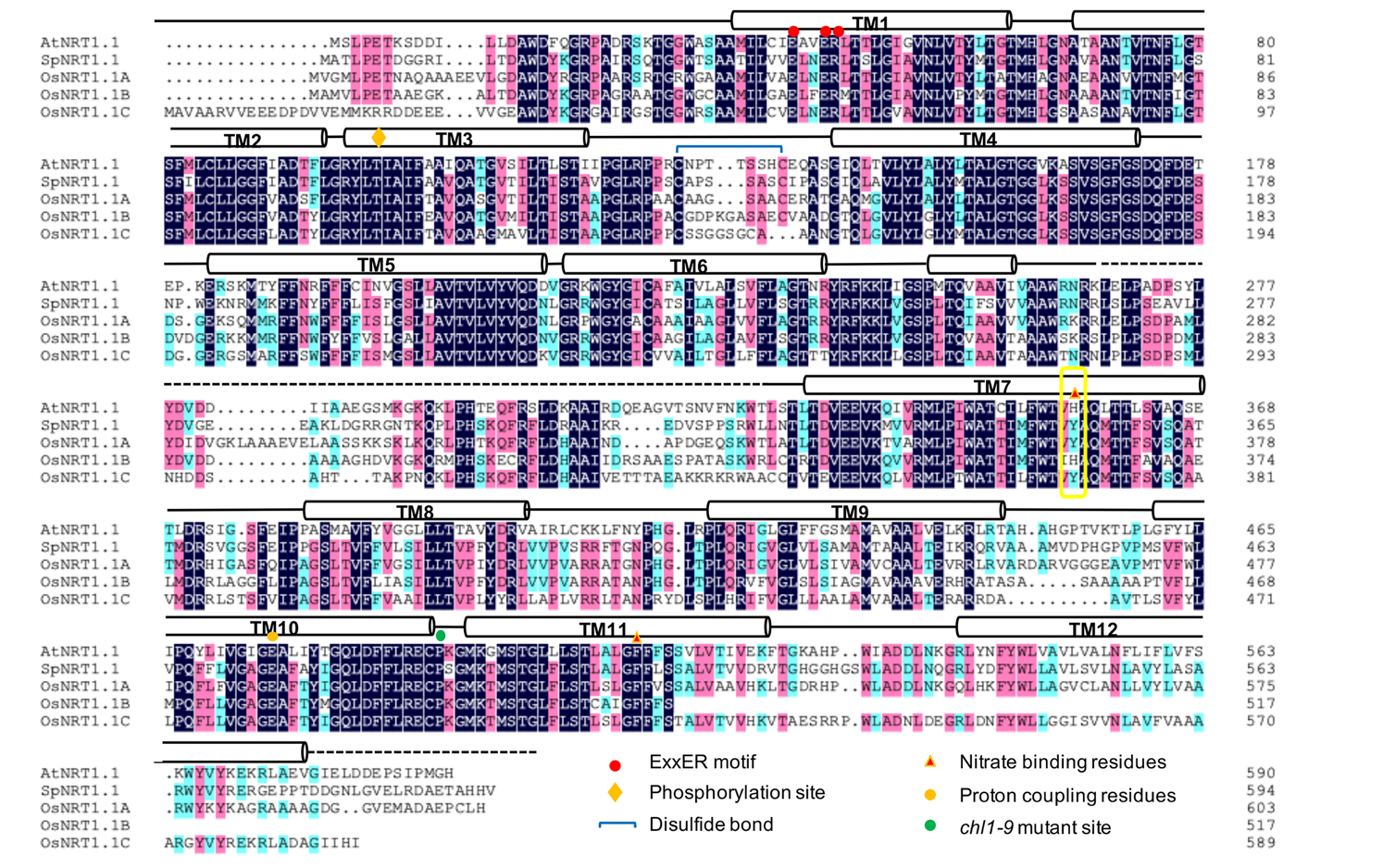


**Supplementary Figure 4. Mulit-alignment between SpNRT1.1 and AtNRT1.1, OsNRT1.1A, OsNRT1.1B, OsNRT1.1C.**

DNAMAN program was used to perform the sequence alignment. The identical residues were shaded in different colors according to identity score, 100% in dark blue, ≥75% in red, ≥50% in wathet blue, <33% in white. The putative transmembrane domains (TM) are marked with blue column and are numbered. The putative ExxER motif marked with green rectangular. The putative disulfide bond marked with red square brackets. The putative proton coupling residue marked with little orange circle. The putative nitrate binding residues marked with red inverted triangle.


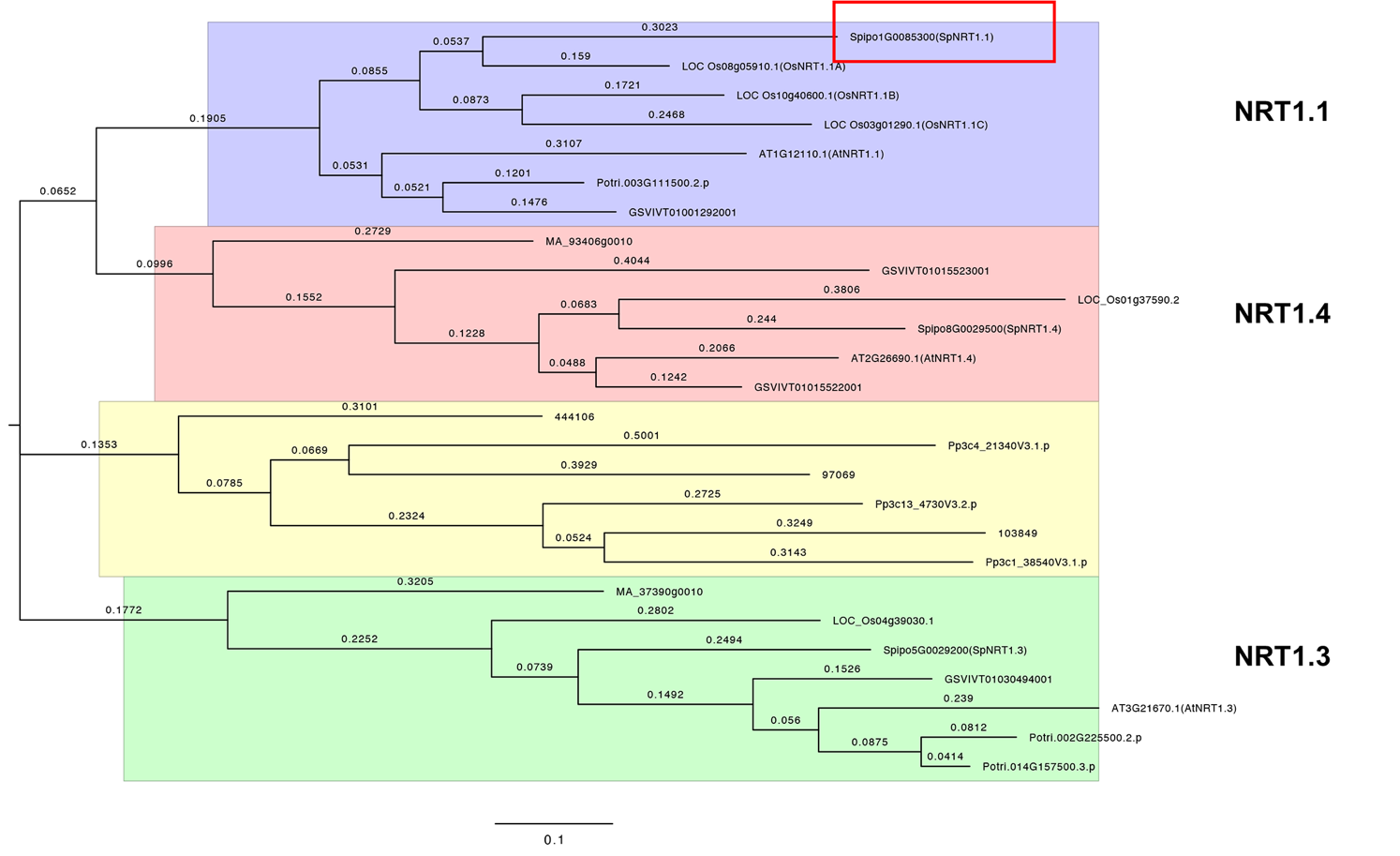


**Supplementary Figure 5. Phylogenetic analysis of NRT1.1 protein in different species.**

The NRT1.1 protein sequence form nonvascular plant *Selaginella moellendorffii* (eg. 444106), *Physcomtrella patens* (Pp3c4_213403.1.p)*,* gymnospermae plant *Picea abies* (eg.MA_93406g0010)*,* and angiospermae plant *Vitis vinifera* (eg. GSVIVT01001292001), *Populus trichocarpa* (eg. Potri.003G111500.2.p)*, Oryza sativa* (eg. LOC Os_08g05910.1)*, Arabidopsis thaliana* (eg.AT1G12110) and *Spirodela polyhiza* (eg. Spipo1G0085300)*.* The different group shaded in different colors, NRT1.1 group with purple, NRT1.3 group with green, NRT1.4 group with red and the other group with yellow.


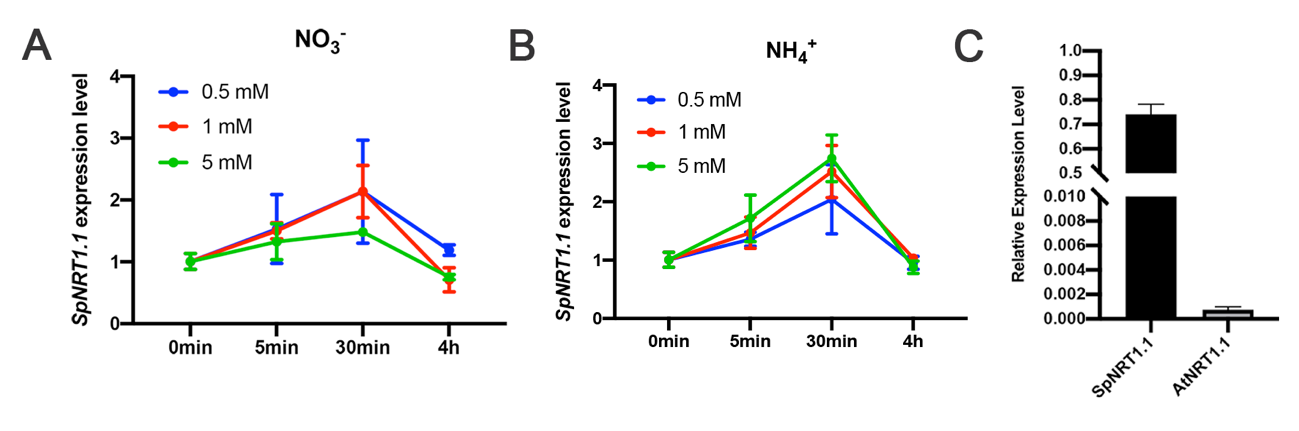


**Supplementary Figure 6. SpNRT1.1 transcript expression is induced by nitrate and ammonium in *Spirodela polyrhiza***

*SpNRT1.1* gene relative expression level measured by q-PCR method in *Spirodela polyhiza* seeding. **(A)** SpNRT1.1 expression level induced by nitrate. **(B)** SpNRT1.1 expression level induced by ammonium. n≥3. **(C)** *SpNRT1.1* relative expression level in *S. polyrhiza* and *AtNRT1.1* relative expression level in *A. thaliana*. The seedling cultured under 5 mM NO_3_^-^ condition.


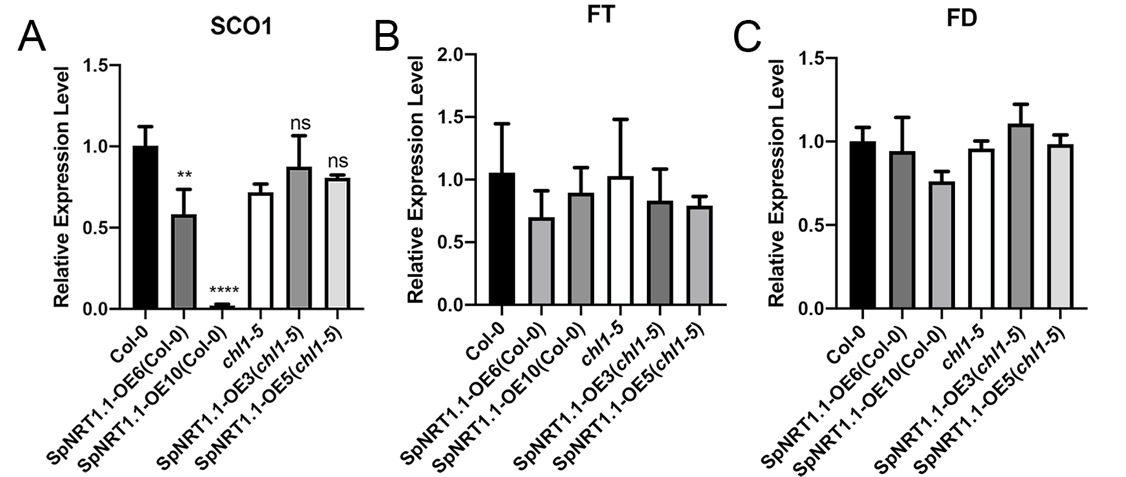


**Supplementary Figure 7. SpNRT1.1 overexpression in Arabidopsis (Col-0) decreases the expression levels of flowering genes.** Student’s *t* test method was used for difference signification analysis. Asterisks indicate signification level compare with Col-0 or *chl1-5* plant, p<0.01(**), p<0.0001(****).


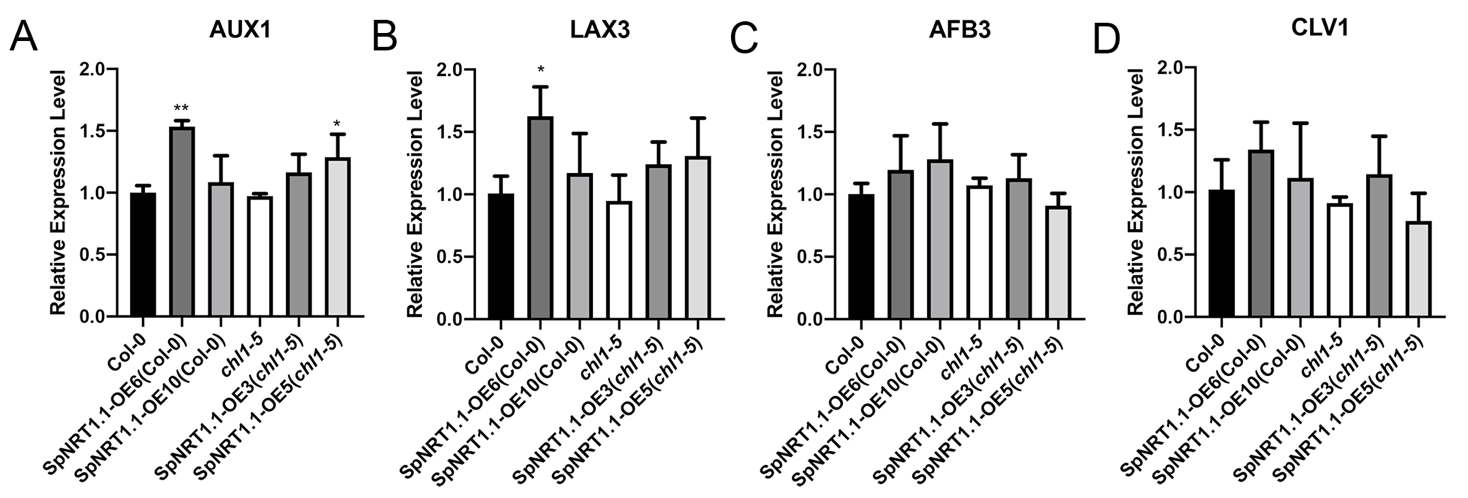


**Supplementary Figure 8. SpNRT1.1 expression in Arabidopsis affect the gene expressions of root development under high nitrogen condition.** Student’s *t* test method was used for difference signification analysis. Asterisks indicate signification level compare with Col-0 or *chl1-5* plant, p<0.05(*), p<0.01(**).
